# Radiotherapy-Induced Atrial Myxoma: A Case Report and Literature Review

**DOI:** 10.3390/life13071585

**Published:** 2023-07-19

**Authors:** Walid Shalata, Ismaell Massalha, Shlomo Yaron Ishay, Elena Chernomordikova, Ashraf Abu Jama, Keren Rouvinov, Yulia Dudnik, Alexander Yakobson

**Affiliations:** 1 The Legacy Heritage Center & Dr. Larry Norton Institute, Soroka Medical Center, Ben Gurion University, Beer Sheva 84105, Israel; 2 Department of Cardio-Surgery, Soroka Medical Center, Faculty of Health Sciences, Ben Gurion University of the Negev, Beer Sheva 84105, Israel

**Keywords:** lung adenocarcinoma, myxoma, radiotherapy, rare tumor, malignant tumor, case report

## Abstract

In this particular case study, we present a 66-year-old male who was diagnosed with an atrial myxoma eight years after receiving treatment for non-small cell lung cancer. The patient underwent chemo-radiotherapy (mediastinal area) in 2012 to address stage III-A adenocarcinoma of the lung. During follow-up imaging in 2020, a left atrial mass displaying characteristic features of a cardiac myxoma was detected. Upon reviewing a computed tomographic (CT) scan from 2017 within the previously irradiated mediastinal region, the cardiac mass was retrospectively identified. The surgical excision of the cardiac mass was performed, and a subsequent pathological examination confirmed the diagnosis of myxoma. To the best of our knowledge, this is the first reported case of a left atrial myxoma in a patient previously treated for adenocarcinoma of the lung and the first instance of an atrial myxoma occurring in a site that had undergone prior radiation therapy.

## 1. Introduction

Primary cardiac neoplasms are exceedingly rare, occurring in less than 0.02% of individuals during their lifetime [1]. Among these, cardiac myxomas are the most prevalent type, accounting for over half of all primary cardiac tumors [2]. Approximately 75% up to 90%, of cardiac myxomas are located in the left atrium, typically attached to the atrial septum near the fossa ovalis [3]. These tumors originate from mesenchymal cells that produce mucopolysaccharide and immature collagen [4]. Atrial myxomas are associated with a triad of complications, including obstruction, embolism, and constitutional symptoms such as fever and weight loss [5,6]. Echocardiography is the preferred diagnostic method for cardiac tumors, and early surgical resection is the only effective treatment to prevent life-threatening complications resulting from intra-cardiac blood flow obstruction or embolization [7,8]. The prognosis for patients undergoing surgical resection of atrial myxomas is excellent, with operative mortality rates not exceeding 5% and rapid postoperative recovery. Recurrence rates range from 1% to 3% in sporadic cases, 12% in familial cases, and 22% in complex atrial myxomas [5,6]. It has been suggested that minimal tumor manipulation, excision with sufficient margins, and thorough inspection of all heart chambers are crucial measures to prevent tumor recurrence [6]. Lung cancer ranks as the leading cause of cancer-related deaths worldwide and has the potential to metastasize to various parts of the body, including the heart [9,10]. Indeed, lung cancer is responsible for the majority of cardiac metastases (17–26%) [10]. Non-small cell lung cancer (NSCLC) accounts for approximately 85% of all lung cancers [11]. More than 70% of lung cancer diagnoses occur in patients over the age of 70, while it is rare for it to be detected in individuals under the age of 45 (approximately 3% of cases). The significant morbidity and mortality rates linked to NSCLC can be attributed to the fact that over two-thirds of diagnoses are made at advanced stages, resulting in a grim prognosis during the late stages when the disease is typically detected [12,13]. The management of NSCLC is contingent upon both the cancer stage and the patient’s overall health. It is crucial to acknowledge that staging plays a pivotal role in determining the feasibility of tumor resection. Stages I and II denote localized diseases that can be surgically removed without concern for metastasis, whereas resection becomes impracticable in stages IIIB and IV [14,15]. We were unable to find any reports of lung adenocarcinoma spreading to the endocardium. Due to its rarity, a cardiac myxoma can exhibit certain characteristic features that, when present alongside a malignant neoplasm, may lead to misdiagnosis as metastasis. To the best of our knowledge, we are presenting the first reported case of a cardiac myxoma that closely resembled metastases of a malignant tumor in a patient who had received radiotherapeutic treatment for lung adenocarcinoma. Furthermore, this represents the first documented instance of an atrial myxoma occurring in a previously irradiated field.

## 2. Case Study

In January 2012, a 58-year-old male with a history of type 2 diabetes mellitus and a smoking habit of 35 pack-years within the past 16 years was referred to the emergency room by his primary care physician. The patient presented with symptoms of coughing and shortness of breath. There was no previous history of cardiac or other medical conditions, and there was no family history of cancer. The patient also reported experiencing right-sided chest pain that had been persistent for three months, along with a recent weight loss of 4 kg within the last month.

Upon physical examination, decreased breath sounds were noted over the right lung, while the cardiovascular examination, including electrocardiogram and auscultation, appeared normal. Routine laboratory investigations, including a complete blood count and biochemical profile, did not reveal any abnormalities. A chest radiograph displayed a ground-glass opacity in the right upper lung (RUL). As a result, the patient was admitted to the hospital for further evaluation. Subsequent chest CT scan results indicated the presence of a 3 cm mass in the RUL, accompanied by right mediastinal lymphadenopathy characterized by an enlarged node measuring 2 cm in diameter. Positron emission tomography–computed tomography (PET-CT) revealed increased metabolic activity in the RUL, specifically in the 3 cm mass, as well as other areas of uptake in the right mediastinal region and right pulmonary hilum.

To ascertain the origin of the mass, a biopsy was performed under CT guidance, which confirmed histopathologic findings consistent with adenocarcinoma originating from the lung. In order to further investigate the possibility of metastatic disease, a magnetic resonance imaging (MRI) of the head was conducted, which showed no evidence of metastasis in that region.

Based on clinical findings, the presumptive diagnosis was stage T1b N2 M0 (stage 3-A) non-small cell lung cancer. To address the condition, the patient underwent tumor resection through a right upper lobectomy procedure, which resulted in clear margins. Following the surgery, the recommended primary treatment was a combination of chemotherapy and radiotherapy (CRT).

**Figure 1:**
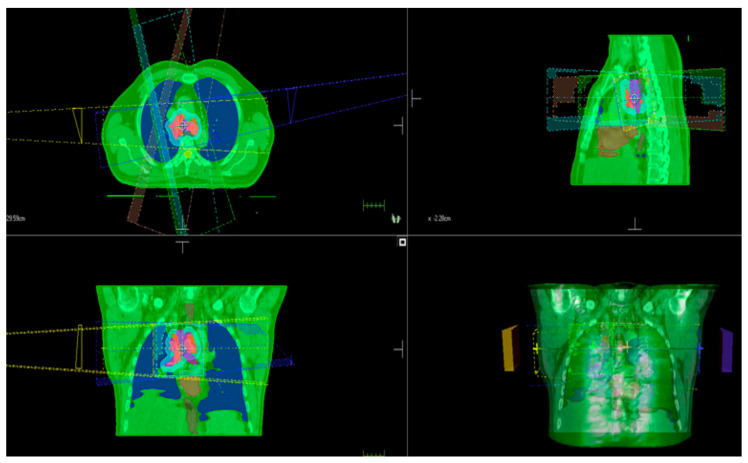
The mediastinal radiation fields.

The patient received sequential CRT, which involved administering vinorelbine at a dose of 30 mg/m^2^ on days 1 and 8 every 21 days, along with cisplatin at a dose of 75 mg/m^2^ on day 1 every 21 days for a total of four cycles. Subsequently, definitive radiotherapy was performed using 3-D CT-guided treatment planning and photon treatment on a linear accelerator. The radiotherapy targeted the mediastinal area, delivering a total dose of 60 Gy in daily fractions of 2 Gy (Figure 1).

During the one-month follow-up examination, chest CT imaging revealed no evidence of disease. After two months of continued follow-up, the major symptoms experienced by the patient, such as coughing and dyspnea, were completely resolved. Further monitoring was conducted through 2019, involving various imaging studies, including MRI of the head, CT of the chest, and PET-CT scans, all of which demonstrated no signs of disease.

**Figure 2:**
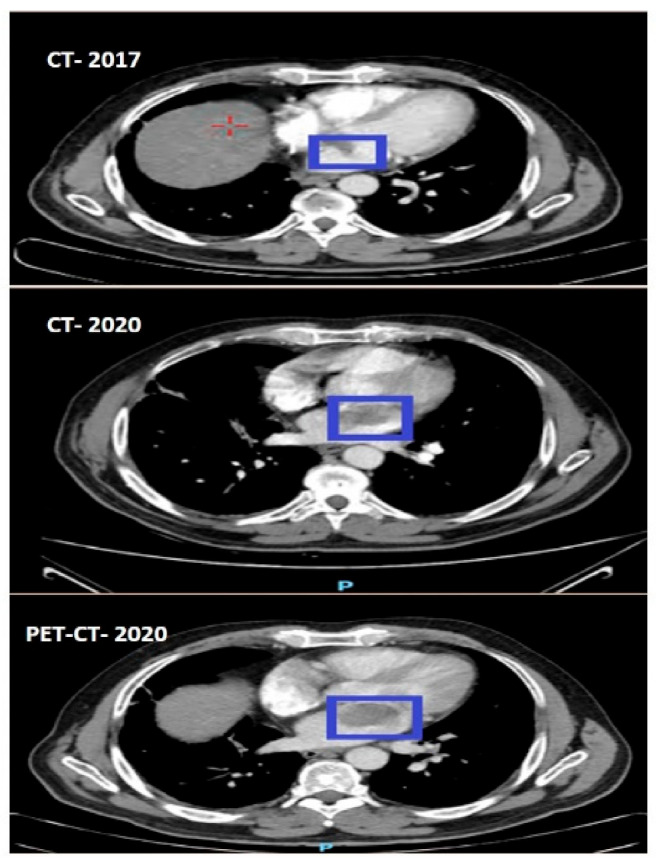
The progressive growth of the myxoma (blue squares) from 2017 until its subsequent removal.

In February 2020, during a follow-up, a chest CT scan revealed a suspicious lymph node in the left side of the mediastinum, measuring approximately 2 cm in diameter, as well as a mass in the left atrium. A month later, a total body PET-CT scan was performed, which showed slight hypermetabolic activity in the suspicious lymph node, diffuse pulmonary nodules with the largest diameter up to 5 mm, and a mass in the left atrium. A multidisciplinary conference involving specialists from various fields concluded that the atrial mass was likely a myxoma. Excision of the mass was recommended instead of observation. Upon reviewing previous CT and PET-CT scans (Figure 2), it was observed that the mass could be seen on the CT scan from December 2017 and had increased in size on subsequent scans. The cardiac mass was situated within the previously radiated mediastinal field. Other abnormalities detected were not indicative of recurrent disease. Close monitoring was advised to detect any potential recurrences or additional primary tumors.

**Figure 3:**
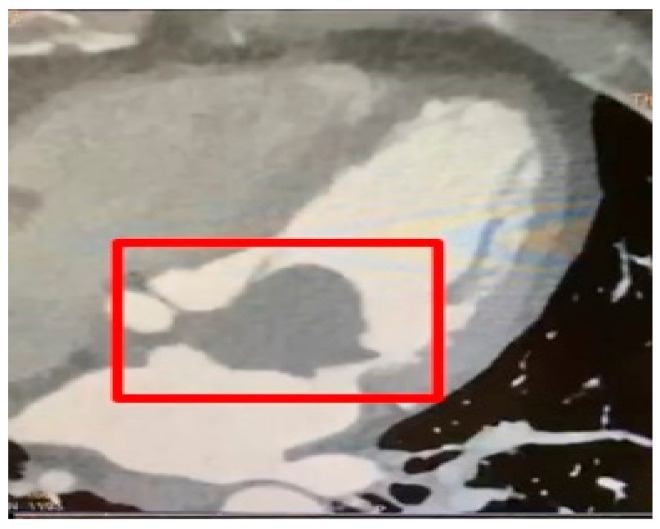
The echocardiogram test revealed a large left atrial mass (red square) measuring 3.6 × 4.2 cm which was attached to the interatrial septum.

Subsequently, the patient was admitted to the cardiology department for further diagnostic evaluations. Electrocardiography showed normal sinus rhythm, while the echocardiogram revealed a large left atrial mass measuring 3.6 × 4.2 cm, highly suspicious for a myxoma, attached to the inter-atrial septum (Figure 3). Mild mitral stenosis, normal left ventricle systolic function with an ejection fraction of 60%, good right ventricle function, mild to moderate tricuspid regurgitation, and severe pulmonary hypertension were also observed. Coronary angiography demonstrated normal coronary arteries.

**Figure 4:**
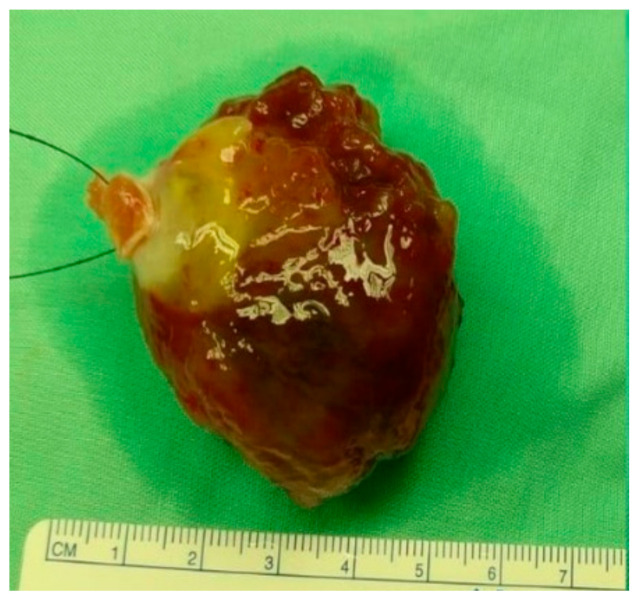
The excised myxoma specimen.

On April 6, 2020, the patient underwent total excision of the cardiac mass in the cardiothoracic surgery department, and the procedure was completed without any complications (Figure 4). A pathologic examination confirmed the diagnosis of myxoma. As of the last follow-up in June 2023, the patient has not experienced any complications related to the radiotherapy treatment, and no recurrence of the myxoma has been observed.

## 3. Discussion and Conclusions

In this case, we have presented a patient who was diagnosed with NSCLC (which is known to be the most prevalent type of lung cancer) [16]. Later, the patient developed an atrial myxoma, a highly uncommon benign tumor found in the heart. It is important to note that myxomas are not typically associated with other malignancies, whether they are currently active or in remission. Cardiac myxoma is recognized as an extremely rare condition, with an estimated annual occurrence ranging from 0.5 to 1 case per million individuals [17]. The most commonly affected sites for atrial myxoma are as follows: the left atrium accounts for approximately 75–95% of cases, the right atrium for 15–20% of cases, the left ventricle for 3–4% of cases, and the right ventricle for 3–4% of cases [3,17].

**Figure 5:**
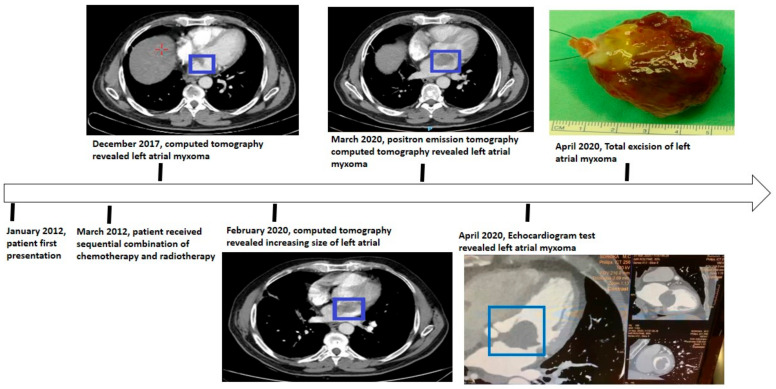
A schematic illustration of the patient timeline from January 2012 (diagnosis of the disease) to April 2020 (the excision of the myxoma (blue square)).

According to our patient, eight years prior to the diagnosis of the atrial myxoma, he had undergone concurrent chemo-radiotherapy (the accepted and recommended approach for stage 3 NSCLC [14]), which resulted in a complete response of the locally advanced lung cancer (Figure 5).

The subsequent evaluation for suspected recurrence of lung cancer revealed the presence of an atrial myxoma within the mediastinal field that had previously received radiation treatment. This highlights the unique nature of the case, as the myxoma was detected in an area that had been subjected to prior radiation therapy.

Regarding the differential diagnosis, trial myxoma can be challenging to distinguish from mural thrombi with myxoid stroma. These two conditions exhibit similar histopathological features, rendering immunohistochemistry testing ineffective in determining differentiation. However, the application of the calretinin marker, which is specific to myxomas, can aid in distinguishing them from mural myxoid thrombi. It is important to note that other malignant tumors, such as primary sarcoma, primary cardiac lymphoma, and large B-cell lymphoma, can also present as mimics of atrial myxomas [18].

Macroscopically, atrial myxomas typically exhibit a pedunculated growth pattern and have a soft texture. Their diameter can range from 1 to 15 cm, and they may weigh between 15 and 180 g. The surface of the tumor can be smooth, villous, or friable. Villous and friable myxomas are more prone to causing embolic events, while smooth myxomas tend to be larger and present with obstructive symptoms [6]. Atrial myxomas are known to secrete vascular endothelial growth factor, which stimulates the formation of new blood vessels (angiogenesis). Additionally, they produce various cytokines and growth factors that can lead to constitutional symptoms, including fever, malaise, anorexia, weight loss, and an elevated sedimentation rate [19].

Atrial myxomas primarily affect females, and the peak incidence is observed between the fourth and sixth decades of life. The gender distribution is not equal, as studies indicate a female-to-male ratio of approximately 2.05:1 for left atrial myxomas and 0.75:1 for right atrial myxomas. It is worth noting that atrial myxomas are relatively rare in pediatric patients, with only a few reported cases in this age group [20,21,22,23].

While the association between the atrial myxoma and previous radiation in this case may be coincidental, there is some basis for speculation regarding a potential correlation. It is postulated that cardiac myxoma cells could arise from adult developmental remnants that are subjected to mitogenic stimuli. Studies have suggested that radiation exposure during radiofrequency ablation for supraventricular tachycardia could slightly increase the lifetime risk of developing neoplasms [24]. Additionally, it has been hypothesized that heart trauma and subsequent local inflammation could contribute to myxoma development [25]. Two potential mitogenic factors associated with radiotherapy that have been suggested to potentially induce the growth of a myxoma are radiation itself and trauma to the heart tissue. The radiation exposure from radiotherapy can have mitogenic effects on cells, promoting their growth and potentially contributing to the development of a myxoma. Additionally, heart tissue trauma caused by the radiotherapy treatment could trigger an inflammatory response, which may also play a role in the formation of a myxoma. These factors highlight the complex interplay between radiation, tissue damage, and the development of cardiac myxomas [18]. In the case of our patient, there were no indications of traumatic, infectious, or degenerative causes, leading us to conclude, through retrospective research, that the etiology was radiation-induced atrial damage. Hence, close follow-up is crucial for patients in remission from malignant tumors, not only to detect primary tumor recurrences but also to identify the development of secondary neoplasms. Cardiac myxomas can be effectively diagnosed using CT and MRI scans, primarily relying on their typical imaging features and location, as mentioned earlier. CT scans reveal that cardiac myxomas usually have a spherical or ovoid shape with smooth or lobular contours. They can be either sessile or pedunculated, as observed in our case. Contrast-enhanced CT scans typically display heterogeneous enhancement, with most myxomas exhibiting lower attenuation compared to the surrounding myocardium. In some cases, calcification may be present, which is more frequently seen in myxomas originating from the right atrium. MRI findings of cardiac myxomas generally show varied signal intensity, reflecting the heterogeneity of the mass’s different components. On T1-weighted imaging, myxomas typically exhibit low to intermediate signals, but these can increase in intensity when there is hemorrhage. On T2-weighted imaging, the intensity can be either low, corresponding to fibrous components, or high, corresponding to the water content of cystic components. When gadolinium contrast is administered for T1-weighted imaging, heterogeneous enhancement is observed, allowing differentiation from a thrombus. Areas of increased vascularity and inflammation are typically associated with increased enhancement, while regions of necrosis may exhibit low signal intensity on post-contrast images.

CT and MRI modalities offer advantages over transthoracic echocardiograms in the diagnosis and investigation of cardiac masses due to their unique imaging characteristics. However, it is important to note that echocardiograms remain readily available and accessible, serving as a valuable tool in the evaluation of cardiac masses [26,27].

To the best of our knowledge, this case represents the first reported occurrence of an atrial myxoma in a previously irradiated field. It underscores the importance of maintaining vigilance and considering the possibility of a new primary tumor, even if it is a rare occurrence, to prevent misdiagnosis as a recurrence of the original malignant disease. Proper evaluation and accurate diagnosis are essential for appropriate management and treatment decisions in such cases.

## Data Availability

Data are contained within the article or are available from the authors upon reasonable request.
